# Methicillin-Resistant Staphylococcus aureus (MRSA) Strikes Deep: Infected Femoral Aneurysm in a Patient With Deep Vein Thrombosis

**DOI:** 10.7759/cureus.86816

**Published:** 2025-06-26

**Authors:** George K Annan, Patrick O Berchie, Alex Kumi, Moises Zouain Estevez, Chinenye Egwuonwu

**Affiliations:** 1 Internal Medicine, Piedmont Athens Regional Medical Center, Athens, USA

**Keywords:** bacteremia, deep vein thrombosis (dvt), infectious aneurysm, infectious femoral aneurysm, methicillin-resistant staphylococcus aureus bacteremia, mycotic aneurysm, mycotic femoral aneurysm

## Abstract

Infectious (mycotic) aneurysms are rare but potentially life-threatening complications of bacteremia. They account for a small percentage of all aneurysms. Early recognition is essential to prevent catastrophic outcomes.

We report a case of a 53-year-old man with a history of methicillin-resistant *Staphylococcus aureus* (MRSA) nasal abscess who presented with fever, vomiting, and progressive right leg swelling. He was febrile and tachycardic. Labs showed neutrophilic leukocytosis (white cell count of 17.4 x 10⁹/L), elevated C-reactive protein (CRP; 127.4 mg/L), and positive blood cultures for MRSA. Doppler ultrasound revealed acute deep vein thrombosis (DVT) in the right femoral and deep veins. Despite vancomycin, he had persistent bacteremia, and antibiotics were escalated to ceftaroline and daptomycin. Computed tomography (CT) angiography obtained for worsening leg swelling revealed a 6.4 x 6.3 x 7.3 cm right superficial femoral artery aneurysm. He underwent urgent excision, ligation, and bypass, followed by serial surgical washouts.

The infectious aneurysm likely resulted from local vascular inflammation caused by an infected thrombus or hematogenous seeding. Persistent fever and worsening leg swelling despite therapy warranted further imaging. MRSA is a well-documented cause of mycotic aneurysms and requires aggressive treatment. In patients with bacteremia and DVT who deteriorate despite treatment, infectious aneurysm should be considered. Early imaging and multidisciplinary care are crucial in preventing rupture and limb loss.

## Introduction

Infectious (mycotic) aneurysms are rare but potentially life-threatening complications of bacteremia. Bacteremia can lead to arterial wall infection through hematogenous spread, resulting in inflammation and weakening of the vessel wall, which predisposes to aneurysm formation. According to the American Heart Association, mycotic aneurysms are rare overall, and the true incidence is not defined in the general population. The femoral artery is the most involved site among peripheral mycotic aneurysms [1].

When accompanied by deep vein thrombosis (DVT), these cases pose significant diagnostic and therapeutic challenges. If left untreated, an infected aneurysm can rupture, which can lead to a life-threatening hemorrhage [1]. This case highlights the importance of recognizing atypical vascular complications of methicillin-resistant *Staphylococcus aureus* (MRSA) bacteremia and the need for timely interdisciplinary management.

## Case presentation

A 53-year-old man with a history of hypertension, hyperlipidemia, and MRSA nasal abscess presented with a five-day history of right lower extremity pain and swelling, as well as four days of persistent vomiting and high-grade fevers up to 101.7°F. There was no history of trauma, cancer, or known hypercoagulable disorder. He endorsed frequent long-distance travel by road.

**Figure 1:**
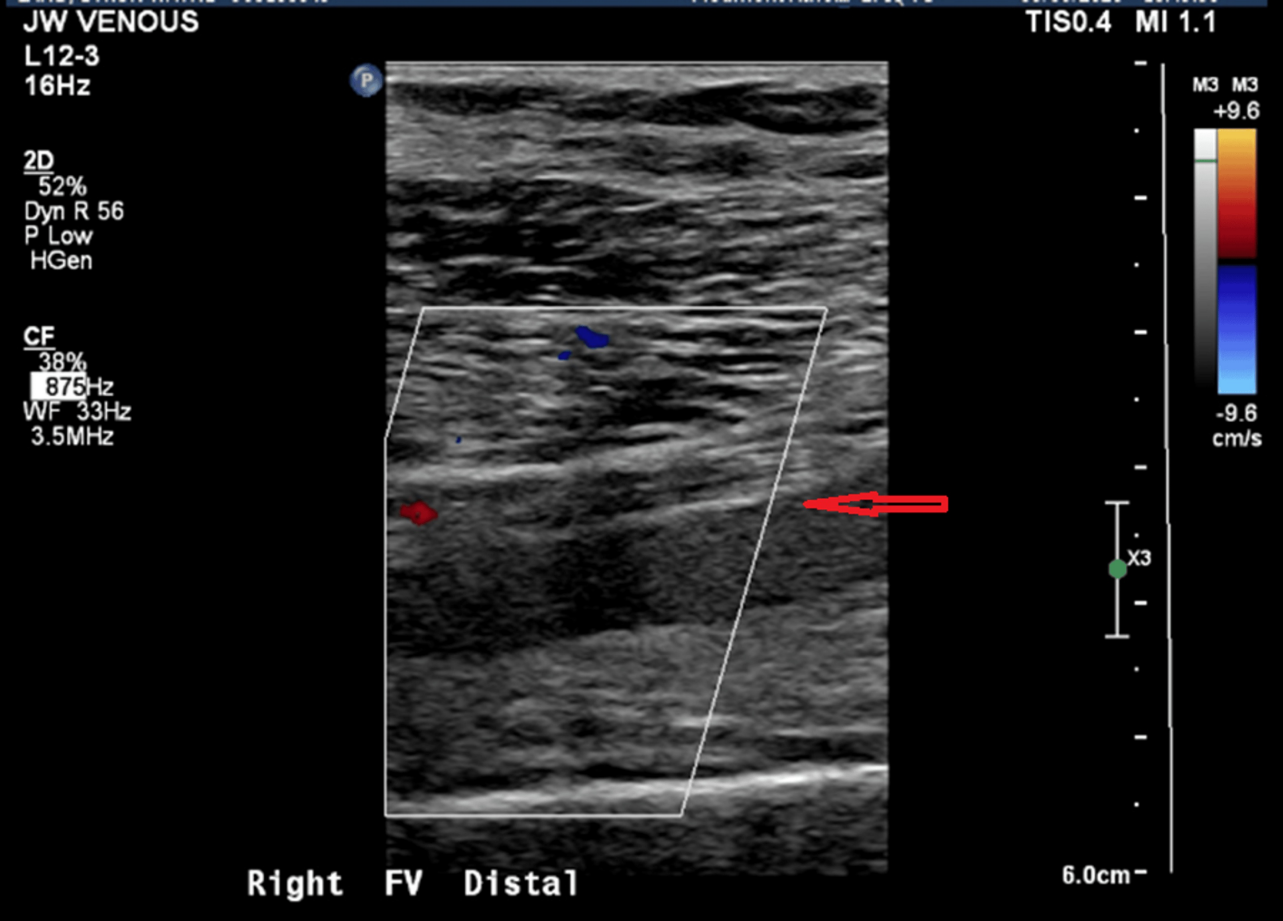
Ultrasound image of right distal femoral vein thrombosis Compression ultrasound of the right thigh showing a non-compressible, dilated right distal femoral vein (arrow) with an acute thrombus, consistent with acute deep vein thrombosis.

**Figure 2:**
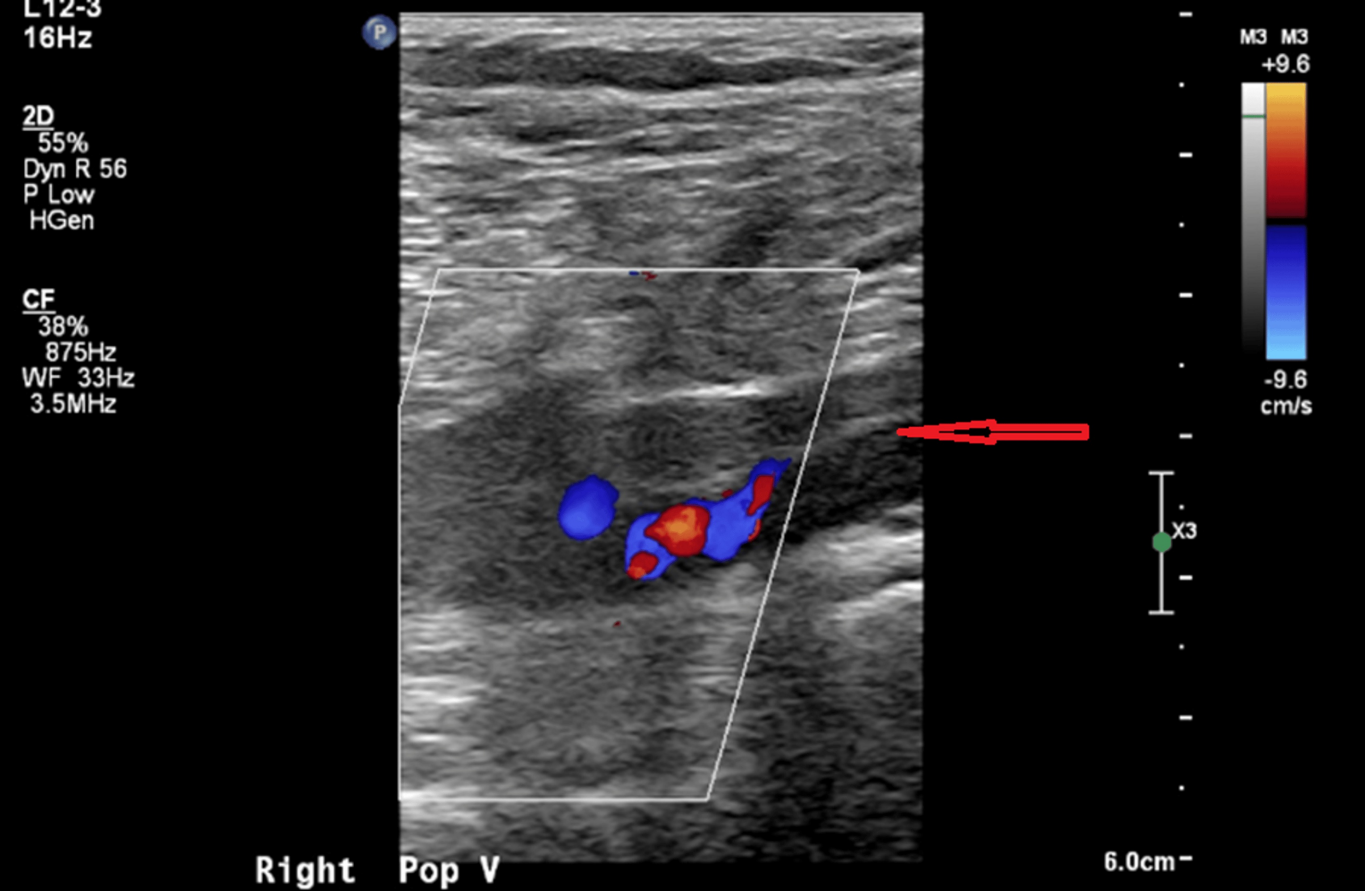
Ultrasound image of right popliteal vein thrombosis Compression ultrasound of the right popliteal fossa showing a non-compressible, dilated right popliteal vein (arrow), consistent with acute deep vein thrombosis.

He was febrile, with a temperature of 100.4°F, tachycardic, and tachypneic. Physical examination was significant for a warm, swollen, and tender right lower extremity. Laboratory workup revealed neutrophilic leukocytosis with a white cell count of 17.40 ×10⁹/L and elevated C-reactive protein (CRP) of 127.4 mg/L. Blood cultures grew MRSA. Venous Doppler (Figures 1, 2) showed acute DVT involving the distal femoral and distal deep venous system of the right lower extremity. The transthoracic echocardiogram was unremarkable.

**Figure 3:**
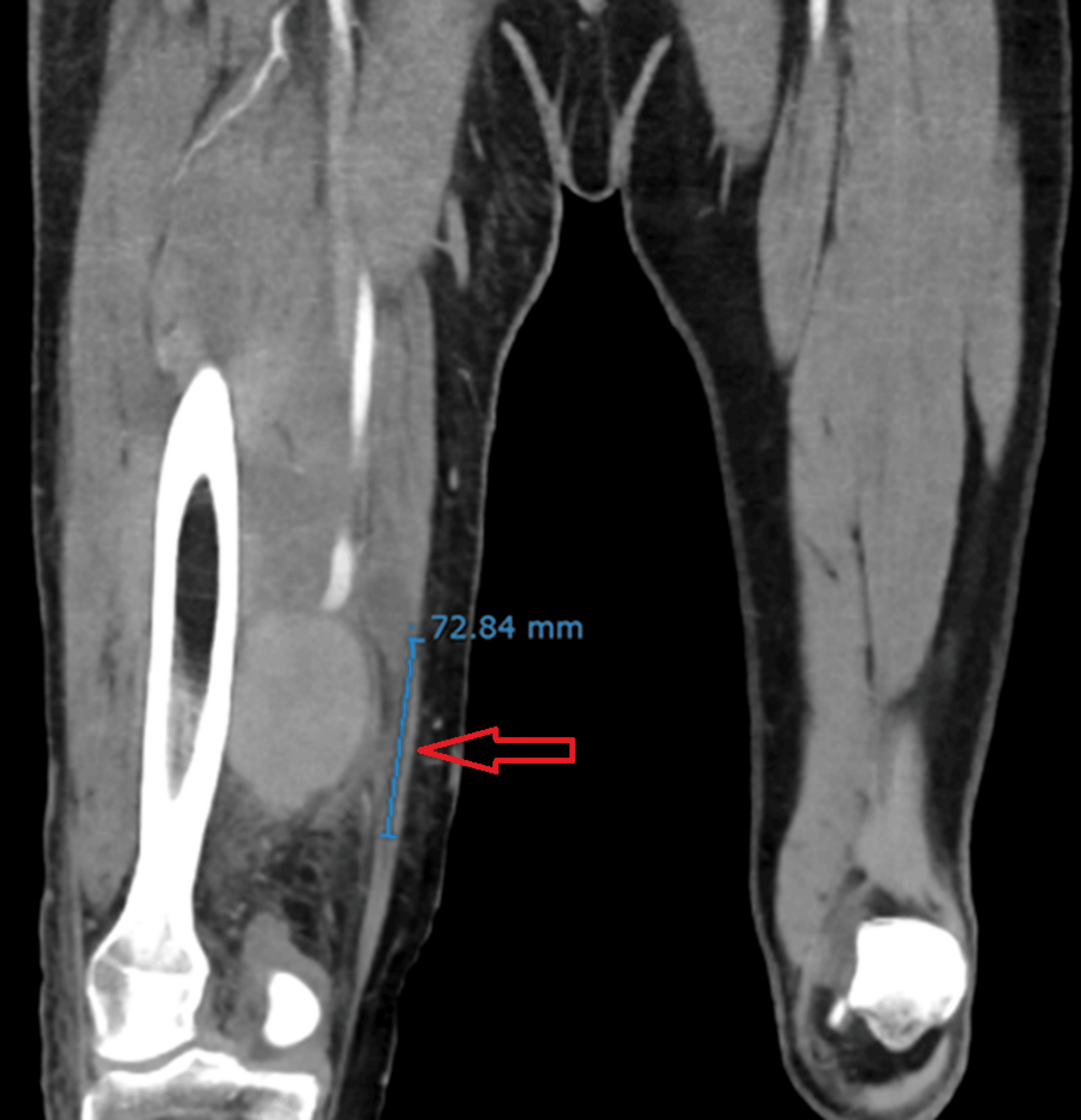
Coronal CT angiogram of right superficial femoral artery aneurysm Contrast-enhanced coronal CT angiography demonstrates a large, saccular aneurysm (red arrow) arising from the right superficial femoral artery, measuring approximately 6.4 × 6.3 × 7.3 cm in cross-section. There is subcutaneous soft tissue stranding in the posterior right thigh and popliteal fossa.

**Figure 4:**
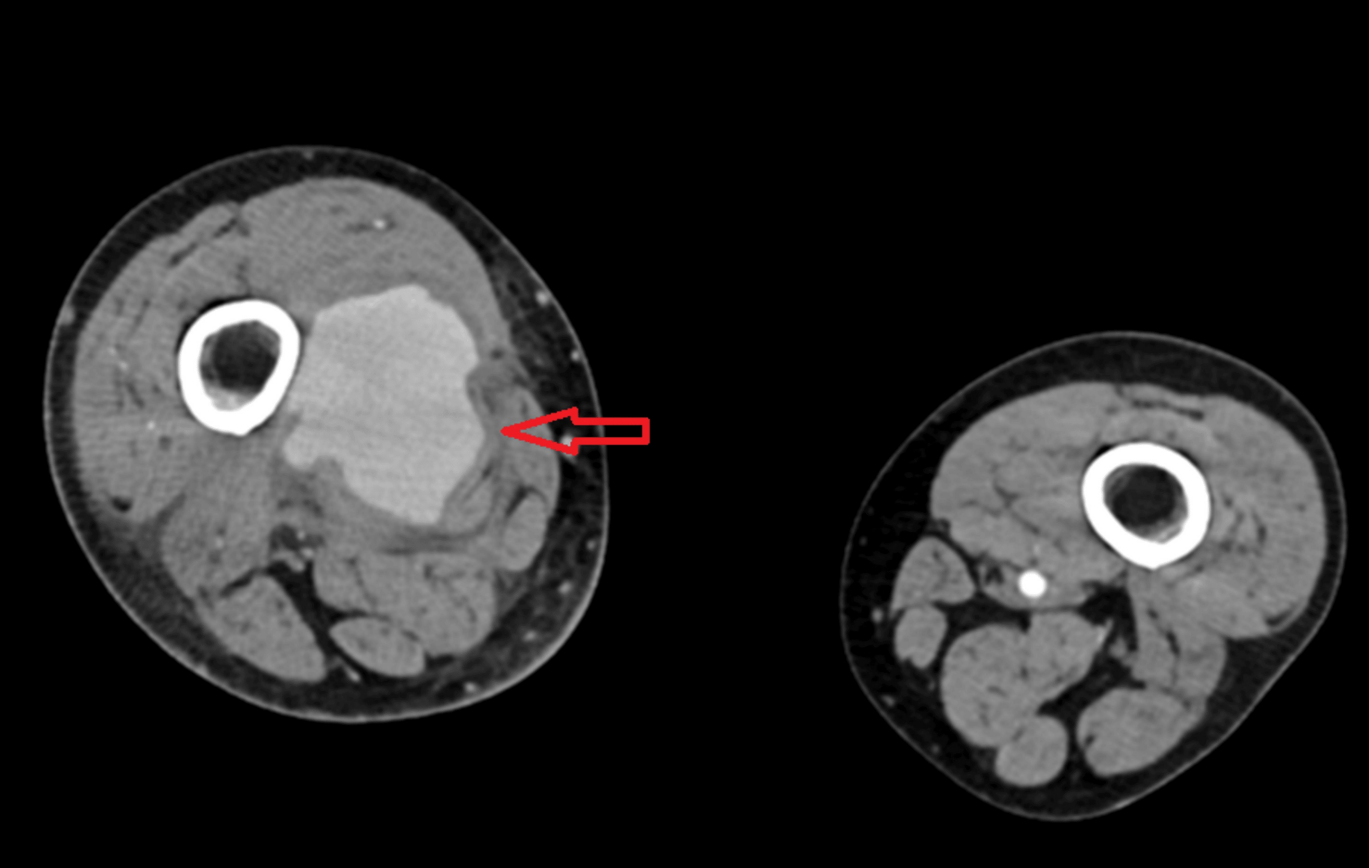
Axial CT angiogram of right superficial femoral artery aneurysm Contrast-enhanced axial CT angiography demonstrates a large, saccular aneurysm (red arrow) arising from the right superficial femoral artery, measuring approximately 6.4 × 6.3 × 7.3 cm in cross-section.

He was initially started on vancomycin and intravenous heparin. However, due to persistent bacteremia and concerns about impaired bacterial clearance from the clot, vancomycin was discontinued, and salvage therapy with ceftaroline and daptomycin was initiated. Despite therapeutic anticoagulation and appropriate antibiotics, the patient had persistent bacteremia and worsening swelling in the right lower extremity. A computed tomography (CT) angiogram was obtained, which showed a large right superficial femoral artery aneurysm measuring 6.4 x 6.3 x 7.3 cm (Figures 3, 4). He underwent urgent surgical excision, ligation, and bypass with subsequent serial washout procedures. The swelling in the right extremity markedly improved with defervescence and resolution of bacteremia. CRP and white cell count normalized. He had a peripherally inserted central catheter (PICC line) placed and was discharged home to continue a total of six weeks of intravenous daptomycin with weekly creatinine kinase monitoring. He was also transitioned to Eliquis. At his two-week follow-up visit, he had no swelling of the right lower extremity or fevers. 

## Discussion

This case highlights an unusual and severe complication of MRSA bacteremia, an infectious femoral artery aneurysm, occurring in conjunction with extensive lower extremity DVT. Infectious aneurysms are rare, representing <5% of all arterial aneurysms, and typically arise from trauma, intravenous drug use (IVDU), arterial catheterization, or contiguous spread from soft tissue infection [1,2]. The triad of DVT, bacteremia, and infectious arterial aneurysm has been reported but mostly in the context of IVDU, where local tissue necrosis, direct vascular injury, and bacteremia create a favorable environment for both venous thrombosis and arterial infection [1].

In contrast to these more recognized etiologies, our patient had no history of invasive vascular procedures or IVDU, making hematogenous seeding of the arterial wall the most likely mechanism, potentially facilitated by adjacent inflammatory compromise from the infected thrombus. The most implicated pathogens are *Staphylococcus aureus* (including MRSA) and, less commonly, Gram-negative bacilli such as *Salmonella *species [1,3,4]. Peripheral mycotic aneurysms, including those of the femoral artery as seen in our patient, are less common than aortic mycotic aneurysms. Precise prevalence data are limited due to their rarity and underdiagnosis [1,5].

Ceftaroline plus daptomycin as salvage therapy in MRSA bacteremia is supported by clinical and preclinical evidence, particularly in cases of persistent or refractory bacteremia after failure of standard therapy with vancomycin or daptomycin monotherapy, with trends towards early bacterial clearance [6-8].

The patient’s evolving symptoms and persistent fever despite therapeutic anticoagulation and antibiotics prompted advanced vascular imaging, leading to timely diagnosis and intervention, averting limb loss and life-threatening hemorrhage. This case illustrates that achieving source control in infected aneurysms may require multiple surgical interventions and that aggressive operative management is an essential complement to targeted antibiotic therapy. This case also highlights the critical role of multidisciplinary collaboration, involving internal medicine, infectious disease, and vascular surgery teams, in achieving favorable outcomes through timely surgical debridement with appropriate antimicrobial coverage. 

## Conclusions

In a patient diagnosed with bacteremia and DVT on therapeutic anticoagulation and antibiotics, progressive limb swelling and persistent bacteremia should prompt the clinician to consider infectious aneurysm in the differential diagnosis. Early vascular imaging, prompt surgical intervention, and coordinated multidisciplinary care are critical to preventing catastrophic outcomes such as hemorrhage or limb loss.
